# Development of Phantom Limb Pain after Femoral Nerve Block

**DOI:** 10.1155/2014/238453

**Published:** 2014-04-28

**Authors:** Sadiah Siddiqui, Anthony N. Sifonios, Vanny Le, Marc E. Martinez, Jean D. Eloy, Andrew G. Kaufman

**Affiliations:** Department of Anesthesiology, Rutgers-New Jersey Medical School, P.O. Box 1790, MSB-E547, Newark, NJ 07101, USA

## Abstract

Historically, phantom limb pain (PLP) develops in 50–80% of amputees and may arise within days following an amputation for reasons presently not well understood. Our case involves a 29-year-old male with previous surgical amputation who develops PLP after the performance of a femoral nerve block. Although there have been documented cases of reactivation of PLP in amputees after neuraxial technique, there have been no reported events associated with femoral nerve blockade. We base our discussion on the theory that symptoms of phantom limb pain are of neuropathic origin and attempt to elaborate the link between regional anesthesia and PLP. Further investigation and understanding of PLP itself will hopefully uncover a relationship between peripheral nerve blocks targeting an affected limb and the subsequent development of this phenomenon, allowing physicians to take appropriate steps in prevention and treatment.

## 1. Introduction

Following a limb amputation, three types of perceptions should be anticipated: stump pain, phantom sensation, and phantom pain. Stump pain is a nociceptive pain that occurs at the existing stump site due to the release of inflammatory mediators and resultant activation of peripheral nociceptors at the surgical site. Phantom sensation can be defined as a perception that the amputated limb is still attached to the body. It is common early in the course and may resolve over time. Phantom pain is characterized by mild to severe, intermittent or constant, stabbing, shooting, cramping, pins and needles, and/or burning pain in the nonexistent limb that is believed to be mediated by both the peripheral and the central nervous systems [1–3]. It is generally chronic and intractable and rarely disappears with current medical management [4]. Interestingly various studies, albeit inadequately powered prospective randomized and nonrandomized trials, have shown that continuous peripheral nerve blocks for extended periods may actually decrease PLP [1, 2, 5–7]. This seems to stand in stark contrast to our case presentation.

## 2. Description

The patient is a 29-year-old male with no significant past medical history who had a scheduled left-sided, above knee amputation two weeks prior to admission. The patient sustained significant injury to the left leg from a motor vehicle accident prior to two years, resulting in chronic osteomyelitis that was unresponsive to treatment. He reported no peripheral nerve damage with the initial injury and was not on chronic pain medication during this time period. After the initial amputation surgery that was performed under combined epidural and general anesthetic technique, the patient reported only surgical stump site pain with no evidence of phantom sensation or PLP. As an inpatient, he was followed by the acute pain service with a functioning lumbar epidural catheter infusion for three days postoperatively. As an outpatient following the amputation, he was treated with opioid analgesics. Two weeks after the amputation, he returned to the hospital for irrigation and drainage (I&D) of the stump site that had become infected. Following the I&D procedure which was performed under general anesthesia, an ultrasound-guided left-sided femoral nerve block was performed postoperatively with sterile technique using a 5 cm, 18 gauge insulated needle at a depth of 3 cm with stimulation current as low as 0.44 mA. Thirty mL of 0.25% bupivacaine was injected after negative aspiration for blood. Immediately after the left femoral nerve block was performed, the patient reported new onset symptoms consistent with PLP including intermittent, pulsatile pain beginning at the stump site and shooting down to the heel with numbness and tingling in the toes. Opioids provided minimal relief and he was started on gabapentin 600 mg PO q8 hours and amitriptyline 25 mg PO qHS to which his pain was responsive.

## 3. Discussion

The exact mechanism of phantom limb symptoms (PLS) remains unclear but is thought to be multifactorial involving the brain, spinal cord, and peripheral nerves. Reorganization, that is, adjacent afferent fibers essentially “taking over” for the functionally inactivated ones within the areas of the cortex and the dorsal horn (e.g., A*β* fibers which normally synapse in deeper laminae sprouting into pain-modulating laminae I and II) representing the deafferentated limb, is believed to be an integral component [3, 8]. Furthermore, direct nerve damage at the amputation site, leading to regenerative sprouting, ensuing ectopy, and increased sensitivity to stimuli, is also thought to play a role [3, 8–10]. MRI and PET scans have demonstrated activity in the brain corresponding to the deafferented area when a patient experiences PLP [1, 3, 9]. Additionally, the loss of input from the limb to the respective dorsal horn of the spinal cord results in decreased normal brainstem inhibitory impulses (and decreased threshold to evoke activity) which usually serve to prevent the thalamus and somatosensory cortex from becoming overloaded with input. As a result, the dorsal horn neurons display increased autonomous activity, likely further contributing to the pathophysiology [8, 11]. In short, PLP is a central mechanism that focuses on altered central processing of peripheral input [9].

We hypothesize that, in our patient, the actual PLP was due to the placement of the femoral nerve block. By blocking the femoral nerve, there was peripheral sensitization of the amputated sciatic nerve. This bombardment of signals at the sciatic nerve in the periphery leads to the central sensitization of pain, ultimately resulting in PLP in our patient. The placement of the femoral nerve catheter essentially unmasked the symptoms of PLP. After the femoral nerve block, the reduced afferent input coming to the spinal cord from those neurons of the femoral nerve resulted in the unopposed amplified spontaneous discharging of the sciatic nerve. This further goes along with the hypothesis that neighboring intact peripheral nerves sharing spinal cord segments with their damaged counterparts can augment their degree of responsiveness and thus, by removing this “taming” action with the peripheral nerve block, PLS could be elicited [12]. Consequently, given that the patient may have A*β* fibers that have synapsed in the pain-modulating centers of the spinal cord in conjunction with the repeated stimulation from the amputated sciatic nerve, one may conjecture that a “wind-up” like phenomenon occurred after performance of the left femoral nerve block [3, 10].

## 4. Conclusion

Our case involves a 29-year-old male status after a left AKA two weeks prior to admission who presented for an irrigation and drainage of a surgical site infection. The patient developed symptoms consistent with phantom limb pain following placement of an ultrasound-guided femoral nerve block. The temporal relationship between placement of the catheter and development of symptoms seems too convenient to be coincidental but whether there was direct correlation or only mere association is uncertain. We are not advocating either for or against the use of peripheral nerve blocks for analgesia in amputees. As cases in the literature dealing with peripheral nerve block-induced PLP, as well as sufficiently powered studies demonstrating their effectiveness in its treatment, are few and far between. We would simply caution the practitioner to consider the possibility of the development of this adverse condition and weigh the risks and anticipated benefits accordingly. Our purpose here is to sustain interest in the topic via a curious and confounding report that will hopefully provide an impetus for further research and larger studies with the ultimate goal of prevention and appropriate, evidence-based treatment for this patient population. Unfortunately, at present, short and long term effectiveness of treatment with opioids, NMDA receptor antagonists, anticonvulsants, antidepressants, calcitonins, and local anesthetics remains indeterminate [4].
